# Corneal Cross-Linking as Treatment in Pediatric Keratoconus: Comparison of Two Protocols

**DOI:** 10.1155/2021/2659828

**Published:** 2021-11-03

**Authors:** Shira Hed, Ran Matlov Kormas, Sagi Shashar, Boris E. Malyugin, Matthew Boyko, Boris Knyazer

**Affiliations:** ^1^Joyce and Irving Goldman Medical School, Faculty of Health Sciences, Ben-Gurion University of the Negev, Beer-Sheva, Israel; ^2^Department of Ophthalmology, Soroka University Medical Center, Ben-Gurion University of the Negev, Beer-Sheva, Israel; ^3^Clinical Research Center, Soroka University Medical Center, Faculty of Health Sciences, Ben-Gurion University of the Negev, Beer Sheva, Israel; ^4^S. Fyodorov Eye Microsurgery Federal State Institution, Moscow, Russian Federation, Russia; ^5^Division of Anesthesiology and Critical Care, Soroka University Medical Center, Ben-Gurion University of the Negev, Beer-Sheva, Israel

## Abstract

**Introduction:**

Keratoconus is a progressive corneal disease commonly treated by collagen cross-linking (CXL). Accelerated protocols have recently become common. This study sought to compare the outcomes of accelerated and standard CXL in terms of visual acuity, keratometry, and tomographic parameters in pediatric population.

**Methods:**

We retrospectively reviewed the files of pediatric patients who underwent standard and accelerated CXL for keratoconus in our hospital, between October 2014 and March 2018. Changes in uncorrected distance visual acuity (UCDVA), best corrected distance visual acuity (BCDVA), tomographic keratometry parameters (*K*_max_, *K*_steep_, *K*_flat_, *K*_mean_), and endothelial density count (EDC) were assessed before and at 6 and 12 months following treatment. The analysis included intergroup and intragroup comparisons.

**Results:**

This study included 53 eyes (44 patients). Fourteen eyes were treated with standard CXL (S-CXL, 3 mW/cm^2^, 30 min), while 39 underwent accelerated CXL (A-CXL, 9 mW/cm^2^, 10 min). Intergroup comparison found insignificant differences between groups, with the exception of better results for UCDVA in the S-CXL group after 12 months (*P* = 0.03). In this study, there was no significant difference between the two protocols postoperatively in BCDVA, *K*_max_, *K*_mean_, pachymetry, or corneal astigmatism.

**Conclusion:**

A-CXL is as safe and effective as S-CXL for stabilizing progressive keratoconus in pediatric population. Larger-sample-size studies with a longer follow-up time are required. Considering the long-term results of 9 mW A-CXL and its safety and efficacy profile, it should be preferred to S-CXL for reducing treatment time and improving patients' comfort.

## 1. Introduction

Keratoconus is an ectatic disease of the cornea leading to visual impairment [1]. Classically, disease onset occurs at puberty. Recent studies estimate its prevalence to be 0.9%–3.3% [2], while information about prevalence in pediatrics is limited and found to be 1 : 200 and 1 : 25 in different studies [3, 4]. Studies have shown that onset at a younger age results in a more aggressive and progressive course of the disease than adult onset [5]. Léoni-Mesplie et al. found that pediatric patients (age <15 years) were diagnosed as having stage 4 keratoconus almost four times more frequently than adult patients (age >27 years; 27.8% vs. 7.8%, respectively) [6]. Therefore, early diagnosis and stabilization of keratoconus at its initial stage is essential to prevent grave ramifications, such as visual impairment or penetrating keratoplasty [7].

Wollensak et al. were the first to report the standard cross-linking protocol (S-CXL, Dresden protocol) in 2003 [8]. S-CXL, now considered the gold standard, consists of epithelial removal, riboflavin (vitamin B2) instillation, and 30 minutes of ultraviolet A (UV-A, 360–370 nm) irradiation. It leads to a photochemical reaction that induces covalent bonds between collagen fibers of the stroma, collagen, and proteoglycans, which strengthens and stiffens the cornea, halting disease progression [9,10]. In the adult population, Koller et al. showed in a prospective study on 105 eyes that S-CXL halted keratoconus progression after 12 months in 92.4%, and Poli et al. in another prospective study showed stabilization of 89% at six years after treatment in patients with corneal ectasia [11, 12]. S-CXL treatment led to 70% postoperative regression in a prospective study on 23 eyes at a mean follow-up time of 23.2 months [8]. Mazzotta et al. in a 10-year follow-up study that included 62 eyes of patients aged 18 and below found that S-CXL treatment stabilized the disease in nearly 80% and reduced the progression rate to 24% [13].

An accelerated protocol for cross-linking (A-CXL) has been recently become common. It is based on the Bunsen–Roscoe law of reciprocity, which allows treatment time to be shortened by increasing the radiation intensity to deliver the same total energy dose. Shorter treatment time is especially important in children, because it can improve their compliance with treatment [9, 14].

Studies on adults have shown similar outcomes in different corneal parameters between the S-CXL and the A-CXL protocols [15, 16]. In children, S-CXL has been found to be safe and efficient and to lead to stabilization of the disease [13, 14,17–19]. Several A-CXL protocols have been found to be safe and efficient in case series studies on pediatric patients [20–23]. However, only few studies have compared A-CXL and S-CXL in pediatric patients [24–26].

In the current retrospective study, we compared visual acuity, keratometry, and tomographic criteria between the S-CXL and A-CXL protocols in children with keratoconus at 6 and 12 months following the procedure, in order to determine if the A-CXL is as safe and efficient as the S-CXL.

## 2. Materials and Methods

We retrospectively reviewed the files of pediatric patients who underwent S-CXL and A-CXL for keratoconus at the Department of Ophthalmology in Soroka University Medical Center, between October 2014 and March 2018. The diagnosis of keratoconus and its progression was made using a Pentacam tomographer (Pentacam, Oculus, Wetzlar, Germany). The preoperative progression was defined as a 1.5 D increase in the mean keratometric value or 1 D increase in *K*_max_ or a decrease of 5% in central corneal thickness at two consecutive evaluations with Pentacam. Patients with progressive keratoconus were treated with either the S-CXL or A-CXL regimen. All patients were treated with CXL after instillation with isotonic riboflavin. Clinical examination and tomography were performed before and 6 and 12 months after CXL treatment.

We included patients aged 18 years and below diagnosed with progressive keratoconus and treated with CXL who completed 12 months of follow-up after the procedure. We excluded patients with a history of any ocular disease or surgery, *K*_max_ over 68 D, central corneal thickness less than 400 *μ*m, history of recurrent corneal erosion or dystrophies, history of corneal herpes virus infection, history of rheumatological and autoimmune disease, or sensitivity to any of the substance that is used in the CXL procedure.

### 2.1. Surgical Technique

CXL was performed under topical anesthesia, with oxybuprocaine hydrochloride 0.4% eye drops used before the procedure. A 8.0 mm diameter of the central corneal epithelium was removed using a blunt spatula or epithelial peeler. Then, iso-osmolar riboflavin (Medio-Cross 0.1%; Peschke Meditrade GmbH, Huenenberg, Switzerland) was instilled after epithelial removal every 2 minutes for 30 minutes based on the Dresden protocol. UV-A was then irradiated at an intensity of 3 mW/cm^2^ for 30 min (S-CXL) or 9 mW/cm^2^ for 10 min (A-CXL). Riboflavin solution was instilled continuously every 2 minutes during UV-A irradiation. The patient was instructed to fixate on the light source, and adequate centration was constantly monitored by the surgeon. All eyes were bandaged immediately after the procedure.

### 2.2. Postoperative Follow-Up

Follow-up visits were routinely performed at 1 day, 7 days, 1 month, 6 months, and 12 months following CXL. All patients were prescribed topical ofloxacin 0.3% qid for 10 days and topical dexamethasone 0.1% qid for a total of one month, with gradual tapering down. Patients were advised to use preservative-free artificial tears as needed. One day following the procedure, a therapeutic contact lens was placed if corneal erosion was greater than 3 × 3 mm (SoftLens; Bausch & Lomb, 14 mm diameter and a base curve of 8.6 mm). The contact lens was removed during the 7-day follow-up. Patients underwent corneal tomography using Pentacam and EDC using Specular (noncontact specular microscopy) both preoperatively and at the 12-month follow-up.

### 2.3. Study Protocol and Data Collection

The study protocol was approved by the Institutional Review Board of “Soroka University Medical Center,” Beer-Sheva, Israel, and adhered to the tenets of the Declaration of Helsinki. Data regarding patients with keratoconus were retrospectively collected from medical reports in outpatient clinics. Keratometry and tomographic parameters were acquired using a rotating Scheimpflug tomography camera (Pentacam HR; Oculus, Wetzlar, Germany). EDC was measured with automated specular microscopy (CEM-530, Nidek Co., Aichi, Japan). Visual acuity parameters were collected using a Snellen chart and converted into logMAR for statistical analysis. BCDVA parameters were collected for patients that use glasses or contact lens and were fitted after the CXL procedure. Demographic parameters (ethnic origin, gender, age, etc.) were extracted from patient files.

### 2.4. Main Outcome Measures

The primary outcome measure in this study was *K*_max_ progression (defined as an increase of 1 D or more in *K*_max_) and regression (defined as a decrease of 1–D or more in *K*_max_), at 12 months following the procedure. The secondary outcome measures included UCDVA, BCDVA, corneal tomographic parameters, minimum corneal thickness at the last visit, EDC values, and postoperative keratoconus.

### 2.5. Statistical Analysis

First, we described the demographic differences between the two groups. Age was described by the mean and standard deviation, and the comparison was performed by the *T* test. The categorical characteristics were described as frequency and percent and analyzed using a chi-square test. In addition, for the primary outcome, we compared between the groups the frequency of patients with *K*_max_ < 1 D (improvement) and patients with *K*_max_ >1 D (progression), at 6 and 12 months after treatment using a chi-square test. A *P* value < 0.05 was considered significant, and all analyses were conducted using SPSS version 24.

To assess differences between the study groups, we compared the eye characteristic changes (deltas) between them (intergroup). Results were described by the mean and standard deviation and analyzed by the unpaired *T* tests with a threshold for significance at *α* = 0.05. In addition, to assess either an improvement or worsening following the intervention, we compared the measured parameters before and at multiple points after intervention within each study group (intragroup), before versus 6 months after treatment and before versus 12 months after treatment, using a paired *T* test with a threshold for significance at *α* = 0.05.

## 3. Results

The study included 53 eyes of 44 patients. Fourteen eyes (13 patients) received S-CXL treatment (26%), while 39 eyes (34 patients) received A-CXL treatment (74%). The mean age of the patients was 15.4 years in the S-CXL group and 14.9 years in the A-CXL group. Fifty percent of the S-CXL group and 59% of the A-CXL group were males. Patients' demographics and clinical characteristics are shown in Table 1. There were no significant differences between the groups.

**Figure 1:**
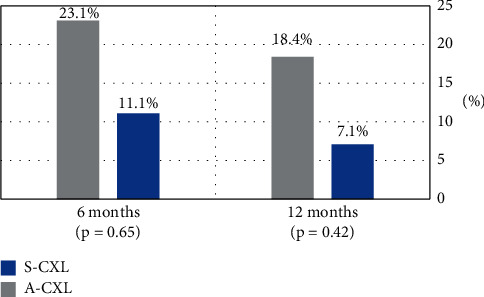
Progression of keratoconus in the study population at 6 and 12 months after CXL. Progression is defined as an increase of >1 D in *K*_max_. A-CXL: accelerated cross-linking; S-CXL: standard cross-linking; D: diopter; KC: keratoconus.

**Figure 2:**
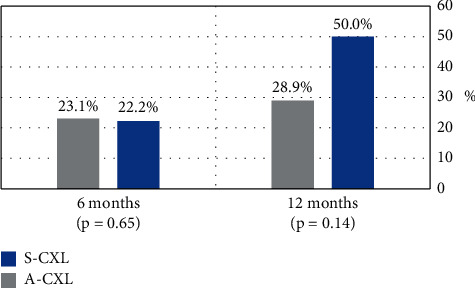
Regression of keratoconus in the study population at 6 and 12 months after CXL. Regression is defined as a reduction of > 1 D in *K*_max._. A-CXL: accelerated cross-linking; S-CXL: standard cross-linking; D: diopter; KC: keratoconus.

Progression in *K*_max_ was observed after 12 months in 7 (18.4%) eyes of the A-CXL group and in 1 (7.1%) eye of the standard CXL group (*p* = .42, Figure 1). Moreover, improvement in *K*_max_, defined as a reduction in 1 D or more, was found after 12 months in 11 (28.9%) eyes of the A-CXL group and in 7 (50%) eyes of the S-CXL group (*p* = .14, Figure 2).

Table 2 shows the results of intergroup comparison of mean change in all parameters at 6 and 12 months from baseline. We found no significant difference in the mean deltas between the groups, except UCDVA after 12 months (mean change of −0.6 in the S-CXL group and +0.1 in the A-CXL group in logMAR, *P* = .03) and *K1* front after 6 months (mean change of 0.2 and −0.4D in S-CXL and A-CXL groups, respectively, *P* = .03).

Intragroup comparison of all variable means before and at 6 and 12 months after the treatment are shown in Table 3. There is no significant difference in visual acuity means after 6 months compared to baseline. However, there was a significant improvement in BCDVA in the A-CXL group after 12 months (reduction from 0.4 to 0.3 logMAR, *P* = 0.04). There was only insignificant improvement in mean UCDVA after 12 months, with better results in the S-CXL group. There was no significant difference from baseline in *K*_mean_, *K*_steep_, and *K*_flat_ for either group. There was also no significant difference from baseline in *K*_max_, but we found a progressive improvement trend in the S-CXL group (reduction of 0.8 D after 1 year), compared to slight elevation (0.2 D) after 6 months and stabilization after 12 months in the A-CXL group. The mean EDC was insignificantly decreased in both groups at 6 and 12 months following the procedure (Table 3). Furthermore, when we compared the deltas of EDC, there was no significant difference between both groups (Table 2). The mean corneal thinnest point reduced significantly after 6 months in the A-CXL group and after 12 months in both groups.

One case in each group was diagnosed as having corneal haze at 1 month following the treatment. They were treated with topical dexamethasone 0.1% with complete resolution by 3 months following treatment, without worsening of preoperative UCDVA or BCDVA.

## 4. Discussion

This study compared the efficacy of S-CXL with A-CXL in pediatric keratoconus patients. We found no significant difference between the two protocols, except in the visual acuity parameter (UCDVA), which was significantly better in the S-CXL group at the 12-month follow-up. Moreover, in most measured parameters, the use of S-CXL showed better keratometric and visual acuity outcomes than of the A-CXL procedure, but these differences were not significant.

Several A-CXL protocols have been found to be safe and efficient, similar to this study. Mazzotta et al. in the recently published “Siena Eye-Cross Study 2” reported long-term results of 5 years of the A-CXL Epi-Off 9 mW/5.4 J/cm^2^ protocol in a prospective nonrandomized cohort of 156 eyes of 112 patients with keratoconus, including 40 eyes of pediatric patients. The study demonstrated statistically significant improvements in UCDVA, BCDVA, *K*_max_, and corneal higher-order aberrations that lasted until the end of follow-up time. Mazzotta et al., in this important report, concluded finally that the 9 mW 5.4 J/cm^2^ A-CXL is the natural evolution of Epi-Off CXL treatment for the management of early progressive corneal ectasia and thus optimizes clinic workflow [23]. Moreover, case series studies on pediatric population found A-CXL to be safe and efficient, but data on this protocol's use in children are still limited and the duration of the irradiation varies [20–22]. Shetty et al. found that an accelerated protocol of 10 minutes is efficient and safe at a 24-month follow-up in patients below 14 years of age [20], while Ozgurhan et al. found that A-CXL for 4 minutes (30 mW/cm^2^) resulted in no disease progression after 24 months [21].

However, only few studies have compared both methods in the pediatric population, with differing results [24–26]. Baenninger et al. performed a retrospective study comparing the standard protocol with an accelerated protocol of 9 mW/cm^2^ for 10 minutes, with a follow-up time of 1 year. The results showed no significant difference between groups [24]. Sarac et al. also performed a retrospective study with a similar accelerated protocol but a longer follow-up time of 2 years; they also found no significant difference between the protocols [25]. The third study, performed by Eissa et al., was prospective and included a shorter accelerated protocol (5 min, 18 mW/cm^2^) with a 36-month follow-up and found a significantly better result in the A-CXL group after 1 year and 36 months in terms of visual acuity, *K*_max_, and manifest refractive spherical equivalent. Notably, while this study compared mean values, the current study compared mean changes [26].

In this study, there was no significant difference between *K*_max_ value of the groups, the primary outcome, similar to the findings of Baenninger et al. and Sarac et al. In contrast, Eissa et al. found a significant difference between the groups in this parameter with better results in the A-CXL group [24–26].

Regarding visual acuity, we found nonsignificant intergroup difference in BCDVA and a significant difference in UCDVA, in favor of the S-CXL group. This differs from other studies, although we found inconsistent results in several studies. Baenninger et al. and Sarac et al. found nonsignificant difference in the changes between groups for both BCDVA and UCDVA [24, 25]. However, Eissa et al. found a significant difference between the groups, with better results in the mean value of UCDVA and BCDVA in the A-CXL group [26]. A possible explanation for the contradicting results of the studies regarding UCDVA and BCDVA may be CXL effectiveness, different protocols of riboflavin instillation and CXL treatment, corneal remodeling, different stages of KC, etc. We believe larger-sample-size studies with a longer follow-up time are required to clarify further aspects of the clinical and imaging outcomes of the A-CXL protocol.

Other keratometric findings (*K*_steep_, *K*_flat_, and *K*_mean_) were also without significant intergroup difference at 12-month follow-up. This is in line with the findings of Sarac et al., who also found no significant difference between groups in simulated keratometry (SimK-1 and SimK-2) after 24 months [25]. In intragroup analysis, they found significant improvement in the A-CXL group, while we found insignificant trends in the means of *K*_steep_, *K*_flat_, and *K*_mean_ for the S-CXL group.

The previous studies of Mazzota et al. and Godefrooij et al. showed that S-CXL treatment was effective in stabilization of keratoconus in 76%–78% in pediatric population [13, 17]. These rates are lower than in the adult population, where stabilization rates are around 90% after S-CXL [17]. The differences between pediatric and adult populations may be explained by several reasons, such as higher rates of atopy and vernal keratoconjunctivitis in pediatric populations, poor compliance, eye rubbing, accelerated corneal collagen turnover in children, and a natural increase in age-related corneal stiffening [2, 13].

In our study, the progression of keratoconus was observed in 18.4% of eyes in the A-CXL group and in 7.1% of eyes in the S-CXL group at 12-month follow-up (*P* = .42). In contrast, Mazzotta et al. in the “Siena Eye-Cross Study 2” reported lower percentage of progression rate (8.33%) in the long-term results after 9 mW A-CXL treatment that occurred within the 2nd and 3rd years. Also, no retreatment was performed after 5 years [23]. Sarac et al. reported a similar progression of 16.3% in the A-CXL group, but a higher progression rate in the S-CXL group (13.1%) [25]. In addition, Baenninger et al. reported a progression of 15.4% in the A-CXL group, but 23.1% in the S-CXL group after 12 months [24]. There are several possible explanations for the higher percentage of progression in our A-CXL group after 12-month follow-up (18.4%) than shown in “Siena Eye-Cross Study 2” findings. One possible explanation is a different technique of riboflavin application. The installation of riboflavin after epithelial removal in our study was every 2 minutes for 30 minutes, based on the classic Dresden protocol. However, in the “Siena Eye-Cross Study 2,” the treatment was performed using the new KXL I system (Averdo, Waltham, USA), which included “presoak” time of only 10 minutes every 1 minute. According to Baiocchi et al., corneal soaking time of 10 minutes after epithelial removal is enough [27] and allows lower rates of cytotoxic free radicals, avoiding excessive corneal dehydration and reducing shield effect that can lessen the CXL penetration and efficacy. Also, it should be noted that the “Siena Eye-Cross Study 2” study population included only around 25% of eyes of pediatric patients. As discussed above, higher stabilization rates in adult population could explain the lower progression rates than in our study. Another possible explanation is the study population. This study included more than 25% patients with vernal keratoconjunctivitis, without a significant difference between the groups. Notably, Baenninger et al. excluded patients with atopy from their study, while Sarac et al. did not collect information regarding vernal keratoconjunctivitis [24,25]. Since the progression rate is known to be higher in patients with vernal keratoconjunctivitis [20], the difference in our S-CXL group compared to other studies may be explained by the lower percentage of patients with vernal keratoconjunctivitis in this group (21.4%) than in the A-CXL group (30.8%).

The corneal demarcation line can be observed by anterior segment optical coherence tomography (AS-OCT) at 14 days postoperatively and may disappear after 3 months. A review by Spadea et al. [28] compared the findings of different studies of corneal stromal demarcation line depth after CXL. After one month, the mean postoperative depth was between 313 and 351 *μ*m after the standard protocol and 288 to 313 *μ*m after the accelerated protocols. The corneal demarcation line is considered to indicate the CXL stromal penetration and efficacy; therefore, superficialization of the treatment by A-CXL may explain the higher progression in this group. Unfortunately, we were not able to perform AS-OCT postoperatively in our study. Furthermore, the retrospective protocol of the study and the fact that the demarcation line is temporary did not enable us to obtain these data.

This study has several limitations. First, it is a retrospective study. Second, both groups have a small sample size. Hence, a larger study will be required to support these results (although it should be noted that previous studies on CXL in pediatric population have included 78 and 87 eyes [24,25]). Third, the follow-up was only 12 months and not longer. Fourth, we used manual data collection, which could potentially lead to some collection mistakes. Finally, anterior segment optical coherence tomography was not routinely performed following CXL; hence, the demarcation line was not examined for either the treatment or control groups.

The study's main strength is the utilization and comparison between multiple parameters and intergroup differences. In addition, the surgical method was identical for all patients in this study, apart from the protocol used (S-CXL versus A-CXL).

## 5. Conclusions

A-CXL is as safe and effective as S-CXL for stabilizing progressive keratoconus in pediatric population. Larger-sample-size studies with a longer follow-up time are required. Considering the long-term results of 9 mW A-CXL and its safety and efficacy profile, it should be preferred to S-CXL for reducing treatment time and improving patients' comfort.

## Figures and Tables

**Table 1:** Demographic and clinical characteristics of the study population.

Characteristics (N, %)	S-CXL (14.26%)	A-CXL (39.74%)	*P* value
Age, years			0.52
Mean ± SD	15.4 + 2.5	14.9 + 2.9	
Median	16.1	15.1	
Min; max	8.8; 18.0	7.6; 18.0	
Males, %	50.0%	59.0%	0.56
Jewish, %	21.4%	20.5%	0.94
Right eye, %	64.3%	38.5%	0.10
Family history of KC, %	57.1%	41.2%	0.48
Vernal keratoconjunctivitis, %	21.4%	30.8%	0.51
Glasses, %	60.0%	46.2%	0.46

SD: standard deviation; S-CXL: standard cross-linking; A-CXL: accelerated cross-linking; KC: keratoconus.

**Table 2:** Intergroup comparison of the delta in outcomes at 6 and 12 months after the procedure.

Variable, mean ± SD	A-CXL	S-CXL	*P* value	A-CXL	S-CXL	*P* value
	Delta after 6 months			Delta after 12 months		
UCDVA (logMAR)	−0.1 ± 0.7	−0.3 ± 0.7	0.66	0.1 ± 0.5	−0.6 ± 0.8	**0.03**
BCDVA (logMAR)	−0.05 ± 0.3	−0.1 ± 0.3	0.79	−0.1 ± 0.3	−0.2 ± 0.2	0.77
EDC (cells/mm^2^)	−18.0 ± 70.0	−21.4 ± 56	0.48	−22.3 ± 11.7	−28.0 ± 9.0	0.34
*K*_max_ (D)	0.4 ± 2.3	−0.3 ± 1.3	0.40	0.2 ± 2.1	−0.7 ± 2.3	0.20
*K1*_flat_ front (D)	−0.4 ± 0.6	0.2 ± 0.8	**0.03**	−0.1 ± 0.9	0.4 ± 1.9	0.29
*K2*_steep_ front (D)	−0.3 ± 1.0	0.2 ± 1.1	0.21	−0.1 ± 1.3	−0.1 ± 1.0	0.95
*K*_mean_ front (D)	0.2 ± 0.8	−0.4 ± 0.8	0.07	−0.1 ± 1	0.2 ± 1.5	0.49
Astigmatism front (D)	−0.1 ± 1.2	0.1 ± 0.5	0.61	−0.04 ± 1.2	−0.2 ± 0.7	0.79

Astigmatism back (D)	−0.1 ± 0.2	−0.1 ± 0.2	0.12	−0.1 ± 0.2	0.7 ± 1.7	0.26
Pachymetry center (*μ*m)	−7.8 ± 22.2	−16.6 ± 16.4	0.29	−7.1 ± 18.6	−3.4 ± 42.5	0.80
Pachymetry thinnest point (*μ*m)	−9.8 ± 23.3	−11.0 ± 21.3	0.90	−11.8 ± 14.6	−29.8 ± 30.2	0.12

SD: standard deviation; delta: value after operation–value before operation; D: diopter; UCDVA: uncorrected distance visual acuity; BCDVA: best corrected distance visual acuity; K: keratometry; EDC: endothelial density count.

**Table 3:** Intragroup comparison of preoperative and 6-month and 12-month postoperative clinical parameters.

Variables	A-CXL					S-CXL				
	Baseline mean ± SD	6-month mean ± SD	*P* value	12-month mean ± SD	*P* value	Baseline mean ± SD	6 months mean ± SD	*P* value	12 months mean ± SD	*P* value
UCDVA (logMAR)	0.7 ± 0.5	0.6 ± 0.5	0.42	0.6 ± 0.5	0.51	0.9 ± 0.6	0.6 ± 0.6	0.40	0.3 ± 0.3	0.11
BCDVA (logMAR)	0.4 ± 0.2	0.4 ± 0.2	0.43	0.3 ± 0.2	**0.04**	0.4 ± 0.2	0.4 ± 0.3	0.54	0.4 ± 0.5	0.11
EDC (cells/mm^2^)	2786.3 ± 172.6	2766.8 + 201.8	0.25	2764.0 + 149.5	0.15	2734.0 ± 171.1	2711 ± 196.3	0.49	2706.3 + 128.4	0.24
*K*_max_ (D)	55.2 ± 4.9	55.4 ± 6.0	0.34	55.4 + 5.3	0.57	54.5 ± 5.5	54.1 ± 4.0	0.58	53.7 + 4.8	0.30
*K1*_flat_ front (D)	46.9 ± 3.1	46.9 ± 3.4	0.15	47.2 + 3.3	0.94	45.9 ± 4.1	45.3 ± 3.0	0.08	45.8 + 4.4	0.47
*K2*_steep_ front (D)	51.0 ± 3.4	46.9 ± 3.4	0.41	51.5 + 3.3	0.76	49.8 ± 4.7	45.3 ± 3.0	0.31	48.9 + 5.1	0.76
*K*_mean_ front (D)	48.9 ± 3.2	48.7 ± 3.6	0.24	49.3 + 3.2	0.63	47.8 ± 4.3	47.4 ± 3.1	0.16	47.7 + 4.6	0.69
Astigmatism front (D)	4.2 ± 1.8	3.8 ± 1.7	0.65	4.3 + 2.1	0.86	3.7 ± 1.9	4.4 ± 1.5	0.55	2.7 + 1.3	0.53
Astigmatism back (D)	1.0 ± 0.4	0.9 ± 0.4	**0.04**	0.9 + 0.4	0.07	0.8 ± 0.4	1.0 ± 0.3	0.43	1.4 + 1.4	0.31
Pachymetry center (*μ*m)	464.7 ± 31.5	462.6 ± 30.4	0.09	457.9 + 42	**0.05**	477.1 ± 45.2	465.8 ± 47.7	**0.02**	471.2 + 70.1	0.81
Pachymetry thinnest point (*μ*m)	460.5 ± 41.1	444.6 ± 33.2	**0.05**	444.7 + 36.6	**<.001**	454.5 ± 46.1	448.0 ± 33.2	0.16	433.8 + 47.7	**0.02**

SD: standard deviation; D: diopter; UCDVA: uncorrected distance visual acuity; BCDVA: best corrected distance visual acuity; K: keratometry; EDC: endothelial density count. *P* values  < 0.05 are considered significant.

## Data Availability

The patients' data used to support the findings of this study are restricted by the Institutional Review Board of “Soroka University Medical Center,” Beer-Sheva, Israel, in order to protect patient privacy. Data are available from the corresponding author for researchers who meet the criteria for access to confidential data.
